# *Neurology*® *Education* Fulfilling an Age Old Mantra in Medicine: Building the Neuroscience Pipeline

**DOI:** 10.1212/NE9.0000000000200011

**Published:** 2022-10-14

**Authors:** Sharon Lewis

**Affiliations:** From the Department of Neurology, University of Pennsylvania, Philadelphia.

See one, do one, teach one. It is this mantra that has been taught in medicine for generations. Fulfilling this approach requires exposure, then proficiency, and finally the gift of paying it forward. When it comes to neuroscience pipeline programs today, the need to pay it forward never rang more true.

By 2025, the shortage of clinical neurologists is expected to be 19%.^1^ Current trends in neurology, neurosurgery, and psychiatry residencies and fellowships do not indicate that a solution is near. One study found that from 2007 to 2018, the percentage of US and international neurology residents has diminished over time.^2^ Early exposure and mentorship are 2 critical ingredients that must be accomplished for students to envision themselves in a neurology career. Students must gain exposure to clinical neuroscience and see how basic science can be applied to a patient with neurologic disease. With exposure, the concept of self-efficacy emerges. Self-efficacy is “an individual's belief in his or her capacity to execute behaviors necessary to produce specific [outcomes],” and “reflects confidence in the ability to exert control over one's own motivation, behavior, and social environment.”^3,4^ This confidence is a predictor of success in science, technology, engineering, and mathematics fields. After achieving a neurology career, the cycle continues; the mentee becomes the mentor, and this perpetuates the pipeline.

This issue of *Neurology® Education* includes 3 articles which describe new innovations to achieve earlier exposure in pipeline programs. These studies demonstrate feasibility in fostering interest in the neurosciences. The article, “Education Research: Bridging the Undergraduate Neurosciences With Clinical Neurology: Neuroscience Faculty Perspectives,” highlights the current landscape.^5^ While neuroscience is the fifth most common undergraduate major for medical students, neurology is considered a viable career path by only 2.7% of students entering medical school.^6^ This study shows that most undergraduate neuroscience courses include discussions about neurologic conditions, but only a few highlight the dearth of neurologists and even fewer opportunities exist for students to engage clinically with neurologists or neurosurgeons—a challenge that has only further worsened with coronavirus disease 2019 pandemic restrictions. In their study, Minen et al. report on results of a survey of 140 neuroscience faculty. The barriers that emerged for these neuroscience faculty were the same as those for students. These include lack of exposure or clinical contacts, inadequate resources, financial hardship and time constraints, regional limitations, and student interest, experience, and discomfort. The most common suggestions for improvement were funding for neurology research experiences, financial support to attend conferences, and connecting with local neurologists. This article also provides suggestions on how to incorporate clinical links into basic neuroscience courses including broader access to clinical journals, opportunities for students and neuroscience faculty to attend national meetings, in-person or virtual shadowing, and opportunities for students to hear what a journey into neurology looks like for faculty. By providing clinical correlation and exposure to practicing physicians, students can envision a career in clinical neurology during their foundational undergraduate years.

Sanderson et al. demonstrate a pathway to early clinical exposure in the article, “Education Research: Enhancing Medical Student Interest in Careers in the Clinical Neurosciences Through a Hands-on Procedure Workshop.”^7^ In this study, preclinical medical students gained exposure to procedural skills in the neurosciences. The hands-on workshop significantly increased interest in the neurosciences. For students who had no interest in neurology, 82% had an increase in interest in the field. This voluntary workshop targeted second-year students suggesting that clinical exposure during the preclerkship years may be an opportune time to excite medical students with procedural aspects of the field.

It is important that while there is a shortage of neurologists in the United States today, the gap in underrepresented neurologists is even more dire. The data are sobering. The Association of American Medical Colleges report, *Altering the Course, Black Males in Medicine*, warns that fewer Black men are applying to US medical schools today than 30 years ago. In 1978, there were 1,410 Black male applicants which declined to only 1,337 in 2014.^8^ The data are no better in residency. The percentage of residents and fellows who are underrepresented in medicine (URM) has decreased since 2011 when compared with the US population. Even when comparing with the percentage of active physicians who are URM, the proportion of underrepresented trainees is lower in neurology residencies and fellowships.^2^ Lack of mentorship and role models play a significant role in the number of underrepresented students pursing medical careers.^9^ The pathway into medicine is arduous. There are many obstacles including financial barriers, negative academic experiences in science gateway courses, feelings of isolation, and bias that may prohibit underrepresented students from pursuing careers in medicine.^10^

Underrepresentation of minority students in medical schools is problematic because diversity improves all students' academic experiences and their ability to work with patients from differing backgrounds.^11^ The article titled “Curriculum Innovations: Creation of a Longitudinal, Neurology-Centered Pipeline Program to Motivate and Support Students From Racial/Ethnically Marginalized Groups” draws on research showing that lack of exposure and mentorship are barriers to pursuing neurology for underrepresented students.^12^ In this study, a pipeline mentorship program provided a cost-efficient approach to overcoming these barriers for undergraduate students. The program uses a tiered mentorship model—similar to residency—with a senior faculty mentor and junior medical students who serve as near peers to the undergraduate students. Clinical exposure was delivered through large group meetings covering professional enhancement topics, clinical cases, shadowing opportunities, and a clinically oriented project. The program provided a staggering 507 contact points over a 2-year period for prehealth undergraduate students who were URM. There was a preference for first-generation low-income students. Sixty percent of participants noted additional benefits aside from the core components including letters of recommendation for summer opportunities and medical school as well as access to research opportunities. Other intangible benefits were achieved and are evident in the narrative comments in Table 5 of the article.

These 3 articles spotlight the shortage of neurologists and practical ways to capture more students' interest in clinical neurology. With the current demand exceeding the supply, there is an onus on the community to deliver programs that increase exposure to clinical neuroscience and share our personal journeys. This is particularly important for recruitment of individuals who are URM. Underrepresented clinicians return to their communities and mentor within the inclusion, diversity, equity and access space, thus coming full circle.^13^ They see one, do one, teach one. However, we must avoid reliance on this diversity tax. The entire neurology community should band together and deliver mentorship to underrepresented individuals to boost the pipeline. With continued research on neuroscience pipelines, we must continue to establish best practices and foster networking and clinical exposure for students. By supporting the pipeline into clinical neurology, we can meet the demand and improve the care of patients.
